# enRoute: dynamic path extraction from biological pathway maps for exploring heterogeneous experimental datasets

**DOI:** 10.1186/1471-2105-14-S19-S3

**Published:** 2013-11-12

**Authors:** Christian Partl, Alexander Lex, Marc Streit, Denis Kalkofen, Karl Kashofer, Dieter Schmalstieg

**Affiliations:** 1 Graz University of Technology, Institute for Computer Graphics and Vision, Inffeldgasse 16, 8010 Graz, Austria; 2 Harvard School of Engineering and Applied Sciences, Visual Computing Group, 33 Oxford Street, MA 02138, Cambridge, US; 3 Johannes Kepler University Linz, Institute of Computer Graphics, Altenberger Straße 69, 4040 Linz, Austria; 4 Medical University of Graz, Institute of Pathology, Auenbruggerplatz 25, 8036 Graz, Austria

## Abstract

Jointly analyzing biological pathway maps and experimental data is critical for understanding how biological processes work in different conditions and why different samples exhibit certain characteristics. This joint analysis, however, poses a significant challenge for visualization. Current techniques are either well suited to visualize large amounts of pathway node attributes, or to represent the topology of the pathway well, but do not accomplish both at the same time. To address this we introduce enRoute, a technique that enables analysts to specify a path of interest in a pathway, extract this path into a separate, linked view, and show detailed experimental data associated with the nodes of this extracted path right next to it. This juxtaposition of the extracted path and the experimental data allows analysts to simultaneously investigate large amounts of potentially heterogeneous data, thereby solving the problem of joint analysis of topology and node attributes. As this approach does not modify the layout of pathway maps, it is compatible with arbitrary graph layouts, including those of hand-crafted, image-based pathway maps. We demonstrate the technique in context of pathways from the KEGG and the Wikipathways databases. We apply experimental data from two public databases, the Cancer Cell Line Encyclopedia (CCLE) and The Cancer Genome Atlas (TCGA) that both contain a wide variety of genomic datasets for a large number of samples. In addition, we make use of a smaller dataset of hepatocellular carcinoma and common xenograft models. To verify the utility of enRoute, domain experts conducted two case studies where they explore data from the CCLE and the hepatocellular carcinoma datasets in the context of relevant pathways.

## Introduction

Biological networks, such as interactions between proteins, biochemical reactions, and signaling processes are commonly depicted in pathway maps. Pathway maps are often hand-crafted and only show the part of the whole known biological network that is immediately relevant for a particular natural process, such as the tyrosine metabolism, or for a particular disease, such as HIV or diabetes. The network described by these pathways is based on published research on the interactions and interdependencies between the various nodes. As a consequence, pathway maps are static and are only valid for the specific processes or disease states they are designed for and fail to adapt to the variation found in real-world data. It is not uncommon, for example, that a de-activation of a node in a cascade invalidates reactions further downstream. For example, the gene *PTEN *is a part of the *phosphoinositide 3-kinase signaling *pathway, which regulates cell-growth [1]. If *PTEN *is mutated it does not fulfill its function and shuts down the pathway, which can lead to tumor growth. Jointly analyzing experimental data and pathways can help in reasoning about and predicting such effects for different conditions. Knowledge about how pathways are modulated by the genetic profile of groups or individual samples can help improving prognosis, treatment, and patient well-being.

**Figure 1:**
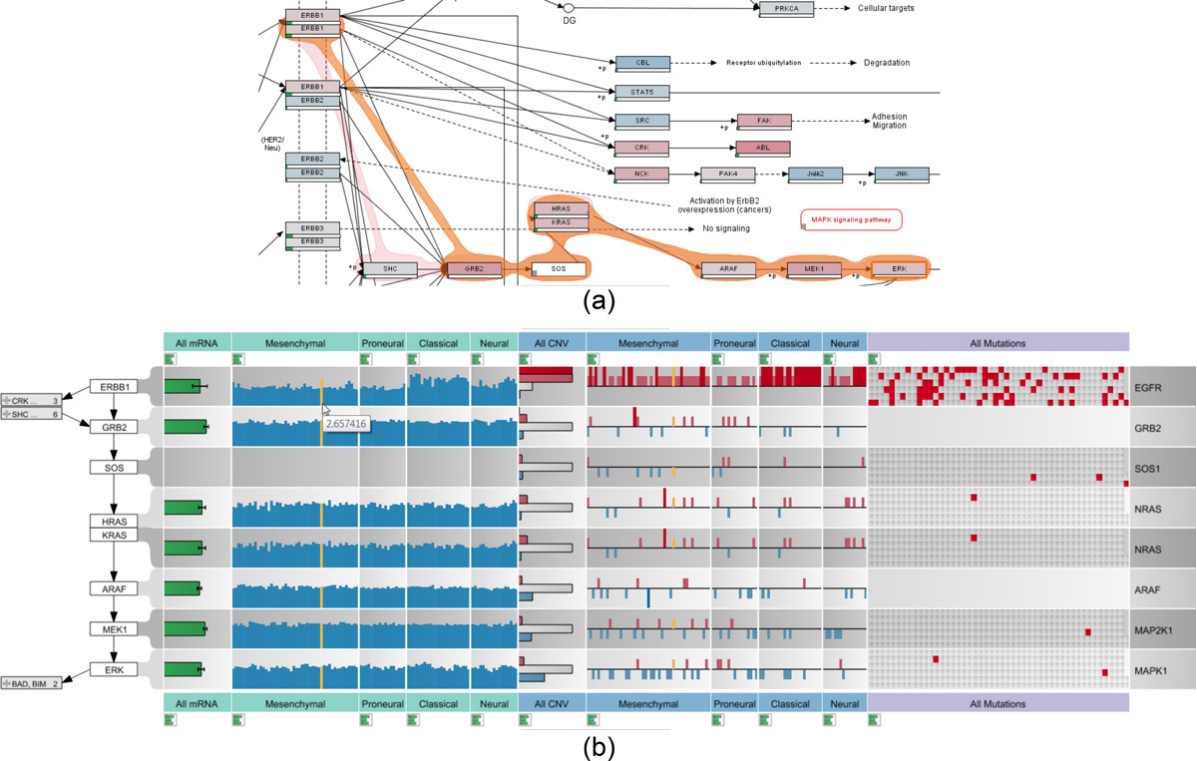
**The dual-view setup of the enRoute visualization technique**. (a) The ErbB signaling pathway from the Wikipathways database, augmented to show abstract experimental data and a selected path (orange). (b) The selected path is extracted and displayed top-down along with associated experimental data from a TCGA glioblastoma multiforme dataset.

Current approaches for visualizing interdependencies between pathways and experimental data do not scale to the now common large and heterogeneous experimental datasets, which often contain hundreds of experiments and multiple data types. We designed enRoute to remedy this. enRoute consists of two views: the pathway view, which shows the whole pathway and hints at interesting paths, and the enRoute view, which visualizes experimental data for parts of the pathway. In the pathway view, shown in Figure 1(a), we show the pathway maps augmented with abstractions of the mapping experimental data. Even though these abstractions are insufficient for an in-depth analysis, they provide an overview and hint at those parts worth investigating in more detail. The enRoute view shows the experimental data for a path that is selected in the pathway view (see Figure 1(a)). The selected path is extracted and juxtaposed with the experimental data, as shown in Figure 1(b). This combined approach successfully addresses the issue of showing large and heterogeneous datasets in the context of networks, the problem of showing multiple groupings of datasets, and it resolves multi-mapping issues that are common in pathway analysis. enRoute is part of *Caleydo*, an open-source biomolecular visualization framework (http://caleydo.org), which features various other visualization techniques for analyzing tabular and network data.

At the beginning of this paper, we give a brief introduction of the biological background, followed by a detailed analysis of the challenges of visualizing graphs with very large numbers of node-attributes. We continue by reviewing the literature and evaluating how existing approaches address the described challenges. Based on this discussion of the state-of-the-art and its limitations, we present our visualization technique, followed by a validation of our approach in case studies, conducted with experts in molecular biology. In the course of these case studies, we demonstrate how enRoute can be used to analyze large datasets in the context of pathways.

This paper is based on and extends previously published work [2]. In addition to a more detailed description of the original concepts, we extend the previous work with a generalization of enRoute to other pathway databases, a novel method to incorporate mutation status data into enRoute, a method to integrate potentially missing edges in the network, a semi-automatic path selection approach, an improved on-node data mapping in pathway maps, and various other improvements. In addition to the conceptual improvements and extensions, we present two new case studies that show the effectiveness of the enRoute technique.

## Biological background

Life scientists have accumulated intricate knowledge about biochemical and signaling processes in living cells, which has been used to build detailed biological interaction networks called pathways. These pathways summarize the molecular interactions in the biochemical conversions of molecules from source material to complex biomolecules or the signaling from a cell surface receptor via a second messenger to the nucleic transcription machinery. Several initiatives are drawing pathway maps and make these maps available to the scientific community, such as KEGG [3] or Wikipathways [4]. In the databases pathway maps are usually categorized by type (e.g., biochemical conversion or signaling pathways) and by biological purpose (e.g., cellular processes, human disease). Biochemical pathways describe the buildup or breakdown of molecules. For example, in the *Glycolysis/Gluconeogenesis *pathway of the KEGG database the buildup or degradation of glucose is described in great detail with all biochemical conversions and the connections to the *Pentosephosphate *pathway and the *Citrate cycle *pathway. A prominent example of the signaling pathway group is the *MAPK signaling *pathway, which contains a well-studied signaling cascade leading from an activated cell surface receptor to a phosphorylation cascade of several kinases to the activation of DNA binding complexes, which regulate transcription of genes involved in the proliferation of cells thus enabling the cell to react to growth stimuli from its environment. It is common for pathways to include the spatial organization of cells like cell walls, the Golgi apparatus, or the cell nucleus, thus allowing to depict transport of molecules and signaling through these compartments.

**Pathway maps **have become a valuable resource for molecular biologists summarizing broad knowledge about molecular interactions and presenting this information in a condensed view highlighting the functional aspects most interesting to the researcher. In the pathway maps nodes represent biological entities like proteins, metabolites, or chemical compounds. Protein nodes are annotated using the gene names from which these proteins are transcribed. These nodes usually contain several isoforms of the same protein and often several proteins of the same family catalyzing the same reaction, leading to extensive multi-mapping of many gene names to a single node. The nodes in a pathway are connected by links that depict biochemical reactions, activating or inhibiting modifications of proteins, or enzymatic reactions. The nodes together with the links between them allow to capture the interaction network of a biological system and present them in a structured graph with node-link diagrams following specific drawing conventions. Most pathway databases contain manually curated pathway maps that are trying to represent the molecular interactions in a visually appealing way while maintaining the scientific content.

In recent years, the advent of high throughput *~*omics technologies has generated large amounts of data. These datasets include genetic and expression data from large consortia like TCGA (http://cancergenome.nih.gov) and ENCODE (http://encodeproject.org/), but also high throughput metabolic screens via nuclear magnetic resonance (NMR) measurements or large scale proteomics by mass spectrometry. Data generated from these analysis includes dynamic data like gene expression levels, metabolite levels, and protein levels in various tissues, cells, and disease states, and also information about the genetic constitution of samples like copy number variation of genes, mutations in genes, or methylation patterns. All this data can only be interpreted in the context of the biological system present in cells, which are captured in the aforementioned pathway maps. The purpose of the techniques presented in this paper is to support the researcher in dynamically mapping biological data onto pathways. This allows researchers to compare, reason, and ultimately explain the complex biological systems and signaling cascades.

## Requirement analysis

As a foundation for the design of our visualization technique and the evaluation of the related work, we have conducted a requirement analysis based on interviews with our collaborators from the Medical University of Graz. Our analysis resulted in five requirements that must be met by a visualization system to successfully enable the joint analysis of pathways and experimental data.

**R I: The Scale Requirement **- While the scalability of the graph is addressed by the sub-division of the biological network into individual pathway maps, the experimental datasets we consider are quite large. Consequently, a joint pathway and experimental data visualization system must be able to scale to dozens of experimental conditions or groups and hundreds of samples.

**R II: The Heterogeneity Requirement **- Modern biological studies often include a wide array of complementary but heterogeneous experimental datasets. While, for example, mRNA expression data measures the gene activity, copy number or mutation data can be used to reason about deviating expression values. These heterogeneous datasets need to be presented using different visualization techniques, as they differ in terms of data type. For example, mRNA expression data is numerical, copy number data is a hybrid categorical/numerical dataset, which is often binned into ordinal (ordered categorical) data, and mutation status data is nominal (unordered categorical). In order to analyze these different kinds of data in context of pathways, the visualization system needs to handle all of them simultaneously and also represent each of them using suitable visual encodings.

**R III: The Multi-Mapping Requirement **- Pathway nodes can represent various different gene products, such as enzymes, proteins, or RNA. In some cases, pathways also summarize a whole gene family into a single node, where different genes produce functionally similar proteins. This is what we call a multi-mapping: One node in a pathway actually represents multiple entities and therefore multiple entries of an experimental dataset can be associated with this node. As understanding this complexity is essential for judging effects of experimental data on a pathway, it is critical to convey multi-mappings adequately.

**R IV: The Layout Constraint Requirement **- Layouts for pathway maps can be either produced automatically or manually. To make pathway maps easier to understand, a number of drawing conventions have been established. Examples are cycles being drawn in circles or the predominance of orthogonal edges in many popular databases. Also, manually curated pathway maps typically contain rich meta-information indicating, for example, the cell compartments in which specific processes occur. Automatically drawn pathway maps can either try to respect these conventions (see, for example, the algorithm by Lambert et al. [5]), or optimize global layout properties using, for example, algorithms for force-directed layouts. We observe that domain experts prefer manual or at least consistent layouts that adhere to these drawing conventions. A reason for that might be that familiar layouts enable them to recognize and understand a pathway only by its topology. While showing experimental data is easier in automatically generated layouts, as the layout can be adapted to suit the representation, a good visualization technique for joint analysis of experimental data and pathways also needs to work with the large baseline of existing, manually produced pathways.

**R V: The Topology-Attribute Coexistence Requirement **- We distinguish between two main types of tasks conducted on a pathway: tasks that are based on the topology of the underlying graph, and tasks that are based on the node or edge attributes of the graph [6]. **Topology-based tasks **are concerned with the connectivity of the graph, e.g., which nodes can be reached from a given node, what are the articulation points of a graph, etc. An example for a topology-based task in pathway analysis is to find all nodes that might be influenced by the inhibition of a node at the beginning of a pathway. **Attribute-based tasks **are concerned with analyzing the properties of node or edge attributes. Edge attributes in pathways commonly describe the type of a relationship between two nodes, such as biochemical conversion, while mapping experimental data represents the majority of node attributes. An example for an attribute-based task for pathway analysis is to find all nodes in a pathway that are mutated in a large number of the mapping samples.

Visualization techniques for graphs are usually optimized for either topology-based task or for attribute-based tasks, but are rarely suitable for both at the same time. Node-link diagrams, are, for example, well-suited for topology-based tasks, while matrix layouts, where nodes are shown on the sides of a matrix and the cells contain information on whether there is an edge connecting the nodes, are ideally suited for edge-attribute-based tasks [7]. When analyzing pathways and experimental data, however, both types of tasks need to be addressed at the same time. The two central questions an analyst is trying to answer when analyzing both pathways and experimental data are (a) how the experimental data for particular experimental conditions or groups of samples influences the topology of the graph and (b) how effects observed in the experimental data can be explained using the topology of the pathways. Consequently, an effective visualization technique has to enable both: an in-depth analysis of the topology and the pathway attributes.

## Related work

While there is a wide body of literature on graph drawing and graph visualization, we focus on the discussion of techniques that are either directly relevant for pathways or that can address the *scale requirement (R I) *with respect to the encoding of node or edge attributes. For a comprehensive review of systems biology visualization refer to the article by Gehlenborg et al. [8]. We identify several techniques that can be used to visualize multiple edge and node attributes in graphs. These are:

• on-node mapping,

• using multiple instances of the graph with different on-node mappings (small multiples),

• using separate linked views for the graph and the attributes, and

• adapting the graph layout.

The benefit of using **on-node mapping **is that it makes it easy to address the *layout constraint requirement (R IV)*. Consequently, on-node mapping has been widely used to augment pathways with multiple colored rectangles, each representing a single experiment or an aggregation of multiple experiments [9-11], where the color encodes the value. There are also variations that use color together with selection and animation [12]. The biggest drawback of this approach is its inability to scale (violating *R I*) as the amount of distinguishable colored rectangles inside a node is severely limited.

An alternative strategy to using multiple colors within each node in one graph is to use multiple graphs where each of them uses a single experiment or a single aggregate of experiments to drive its color-coding. This approach is commonly referred to as **small multiples**. Small multiples show the same configuration of a plot multiple times while changing one variable ([13]pp.170-175). An example that employs small multiples for automatically layouted pathways is Cerebral [14]. Lex et al. have used small multiples to show differences between experimental data associated with cancer subtypes on top of KEGG pathways [15]. Again, scale (*R I*) is a limiting factor. Depending on the pathway about four to ten multiples are reasonable.

A technique that can easily address the scale (*R I*) and the heterogeneity requirement (*R II*) is using **separate linked views **for the experimental data and the pathways. This, of course, also preserves the topology (*R IV*) and can be used to address multi-mappings (*R III*). Separate linked views use synchronized highlighting (linking & brushing) between the multiple views to communicate relationships. If, for instance, a user selects a node in the pathway, the corresponding experimental values are highlighted in the views depicting the experimental data. Shannon et al. [16] and Barsky et al. [14], for example, use a parallel coordinates plot for experimental data, which is linked to a graph depicting protein interaction and metabolic networks. Streit et al. [17] use heat maps and parallel coordinates to show experimental data related to pathways. The major shortcoming of the separate linked views approach is its failure to address *R V*, to simultaneously enable topology-and attribute-based tasks. As separate linked views require interaction to show relationships between a single node and its associated data, the joint analysis of the topology and attributes is severely hindered.

Finally, there are methods that **adapt the graph layout **to be able to show experimental data in pathways. There are numerous systems that calculate an automatic layout for pathways (violating *R IV*) and choose a node size that enables in-place encoding of experimental data with various visual encodings (e.g., bar [18,19] and line charts [20]). While this approach scales a little better than simple on-node encoding, it fails to scale to larger numbers of experimental values (*R I*).

There are also more radical adaption approaches for the graph layout. Schulz et al. [21], for example, use two tables, one for each "side" of a bipartite network and connect the rows in the tables with edges. Each node is represented by one row and there are multiple columns for node attributes. *GraphDice *by Bezerianos et al. [22] probably takes the most extreme approach by laying out the nodes purely based on their node attributes in a scatterplot while still drawing the edges. Both approaches severely hamper the interpretability of the topology, violating *R IV *and *R V*.

A different approach on adapting the graph layout was taken by Meyer et al. with their *Pathline *tool [23]. Pathline uses a linearized version of a pathway where branches and cycles are conveyed using special visual encodings. Next to the linearized pathway the system shows the *Curvemap *view, which displays experimental data for both genes and metabolites recorded in time series. While Pathline was the main inspiration for our approach, it suffers from the unconventional pathway layout, which can hinder understanding the graph topology (*R V*). Also, it currently requires manual creation of the linearized pathways, thereby making it difficult to integrate the large existing databases of pathways.

## The enRoute visualization technique

**Figure 2:**
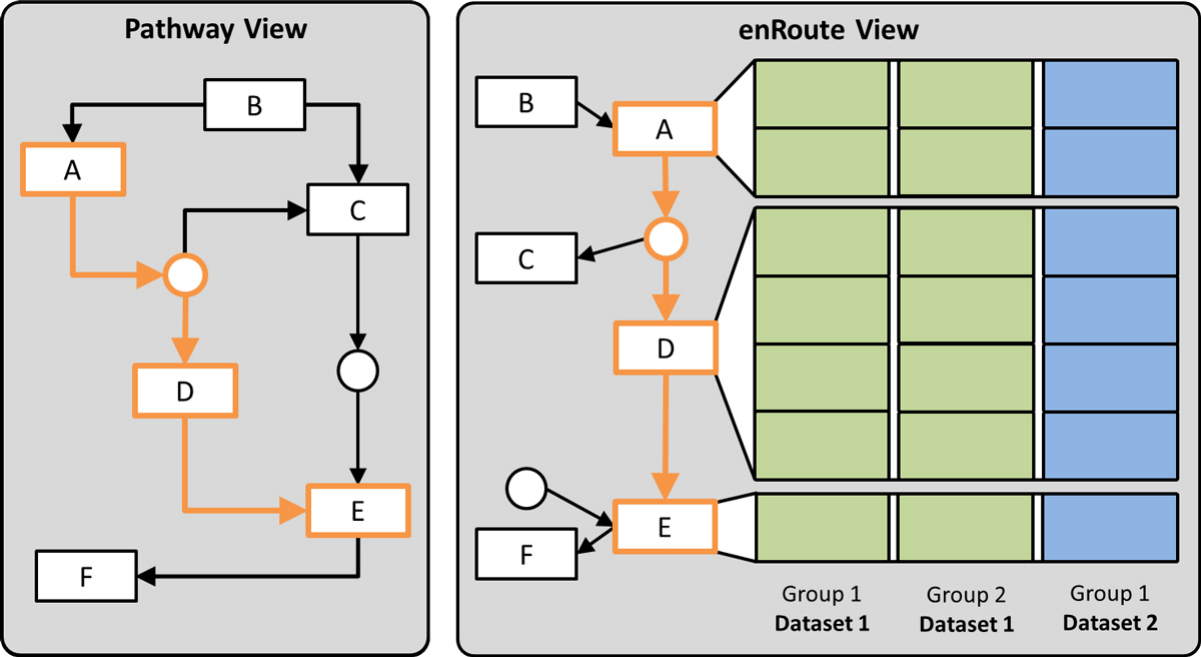
**The enRoute visualization technique with its two basic building blocks: the pathway view and the enRoute view**. The pathway view shows the pathway map in its original layout. In the example shown a path from node *A *to *E *is selected, which is extracted and shown on the right in the enRoute view. Due to the linear layout of the extracted path, the associated experimental data can be visualized next to it. The data can originate from different datasets and can be grouped.

The goal of the enRoute visualization technique is to jointly visualize experimental data and pathways in a way that addresses all five requirements discussed. We identify the topology-attribute coexistence requirement (*R V*) as the most critical requirement to address, as current techniques usually either support only topology-based or attribute-based tasks. Only small-multiples and direct on-node mapping are able to address requirement *R V*, however, both neither scale to many experiments (*R I*), nor do they allow to simultaneously present heterogeneous data (*R II*). Our solution to this problem makes use of an observation we made in discussions with our collaborators: they usually reason about and analyze experimental data associated with a single path at any given time in detail, while the rest of the network merely informs them about the context of this path. They of course continuously change the path of interest, but do not need to see detailed data for multiple paths at the same time. This temporal separation of high-level topology-based tasks and low-level attribute-based tasks allowed us to create a solution that meets all five requirements. The enRoute visualization technique, as depicted in Figure 2, is a dual-view approach consisting of the *pathway view*, showing the pathway map in its original graph layout (meeting *R IV*), and the *enRoute view *where a user-selected path is shown in a linear fashion together with a potentially large number of experimental data from multiple sources (*R I *and *R II*). Due to the linear arrangement of the nodes from top to bottom, it is possible to encode multi-mappings (*R III*) by giving them more vertical space. enRoute thus makes use of the temporal separation of analysis focus by presenting an overview in one and the details of a selected path in another view. In the following, we discuss the components of our approach and their interplay in more detail.

### Pathway view

The pathway view supports two tasks that are an integral part of our approach. First, it is the primary view for conducting topology based tasks. Second, it is used to interactively select the path that is then shown in the accompanying enRoute view along with the associated experimental data. To facilitate identifying interesting paths, the pathway view also shows averages and variances of the mapped experimental datasets. In this section we provide details about our design of the pathway view and its features.

#### Selecting and visualizing the path

An integral part of the pathway view is to allow analysts to determine the path that shall be investigated in the context of experimental data using the enRoute view. In this section we describe methods to select and visualize the paths.

The obvious way for visualizing selected paths in pathway maps is to simply highlight the edges along the path, by, for instance, changing their color or width. Instead of highlighting the edges, however, we decided to use the *Bubble Sets *technique [24] to convey selected paths. Bubble Sets is a method to highlight sets of spatially distributed data points. The elements of each set are wrapped with a continuous iso-contour. We use a slightly modified version of Bubble Sets, as we need to highlight paths instead of sets. Figure 1(a) shows an example of a highlighted path.

Compared to simple edge highlighting, the contour-based Bubble Sets are more salient and can therefore be perceived faster. Furthermore, due to their curve-shaped outline, Bubble Sets can be easier discriminated from the mainly orthogonal structures in the pathway maps [25].

**Figure 3:**
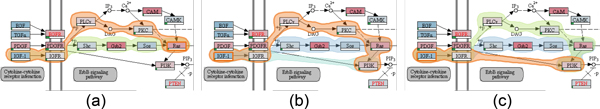
**Multiple differently colored Bubble Sets, each visualizing an alternative path between two user-selected nodes**. In (a) the analyst has selected *IFG-1 *as a start and *Ras *as end node. In (b) the path is extended to also include the *PI3K *gene. This results in a newly added alternative path, which is finally selected by the analyst in (c).

For selecting a path, analysts can choose between two methods: the *iterative approach *and the *start-stop approach*, which can be combined at will. In the iterative approach the analyst can directly select a series of connected nodes that should be part of the path of interest. After selecting an initial node, the analyst can interactively extend the path in both directions by holding the control key while clicking connected nodes. Figure 3(a) shows a selected path in orange, which is extended to include one additional node in Figure 3(b). In the second path selection method, the start-stop approach, analysts pick a start and end node between which all possible alternative paths are highlighted. We use a slightly adapted version of the Bellman-Ford algorithm [26] to find the paths between the two user-selected nodes. The shortest path is selected by default, as shown in orange in Figure 3(a), however, analysts can switch to all possible alternative paths by either using the mouse wheel or by directly clicking a path representation. Figure 3(c) demonstrates a switch to an alternative path with respect to the path selected in Figure 3(b).

While the iterative approach allows analysts to determine paths that cover various kinds of topological structures like, for instance, cycles, the start-stop approach makes it possible to investigate multiple alternative paths between nodes without the need to find and select the route by hand. Additionally, the start-stop approach is more efficient for selecting longer paths.

**Figure 4:**
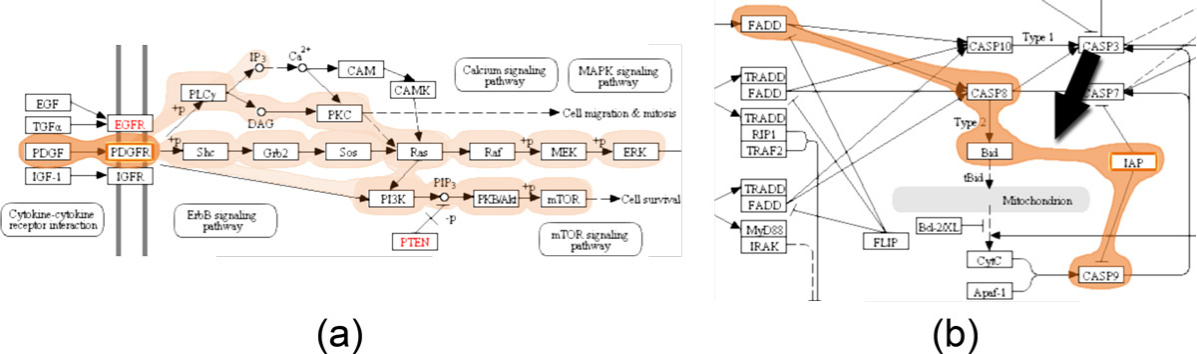
**Support for path selection**. (a) Showing possible extensions of a path using the *preview mode*. Here, all paths continuing after *PDGFR *are highlighted. (b) Adding edges to paths that do not exist in the original pathway. Notice that no edge is shown between *Bid *and *IAP *in the original pathway map, but is introduced using the *force mode*.

However, pathway maps are often very complex and sometimes it is not obvious which choices are available for a path. To address this we provide an interactive *preview mode *for selecting paths on user request. Starting at the end of the current selection, we highlight possible extensions. For example, in Figure 4(a) all edges and nodes are highlighted which extend the end of the current selection at *PDGFR*.

In some cases, the information of pathway maps is not complete or simply outdated. As a consequence, they may not reflect the true process, especially not for all experimental conditions. Additionally, pathway databases can also contain errors that users are aware of. In order to cope with such incomplete or outdated pathway descriptions we provide a *force mode *for selecting paths. This mode enables analysts to add an edge to the pathway, which does not exist in the database. Notice that the second to last edge of the selected path in Figure 4(b) does not exist in the pathway map, neither in the image, nor in the underlying graph representation. By using the force mode during path selection, analysts are able to extend the current path by arbitrary nodes within the pathway map.

#### Visualizing experimental data on pathways

As discussed, directly mapping experimental data on pathway nodes using color-coding does not scale to more than a few experimental values, due to the small size of the nodes in the pathway maps. Despite this limitation, direct on-node mapping is valuable in two scenarios: First, it allows analysts to gain an overview of the main trends in the pathway. Having this overview can be helpful additional information for finding interesting paths. In the second scenario analysts want to investigate a condition (a group of samples) or a single sample in its high-level topological context. This allows analysts to consider experimental data associated with nodes that are not in the currently extracted path. For this purpose, the pathway view can be configured to show only the mapping of selected samples.

**Figure 5:**
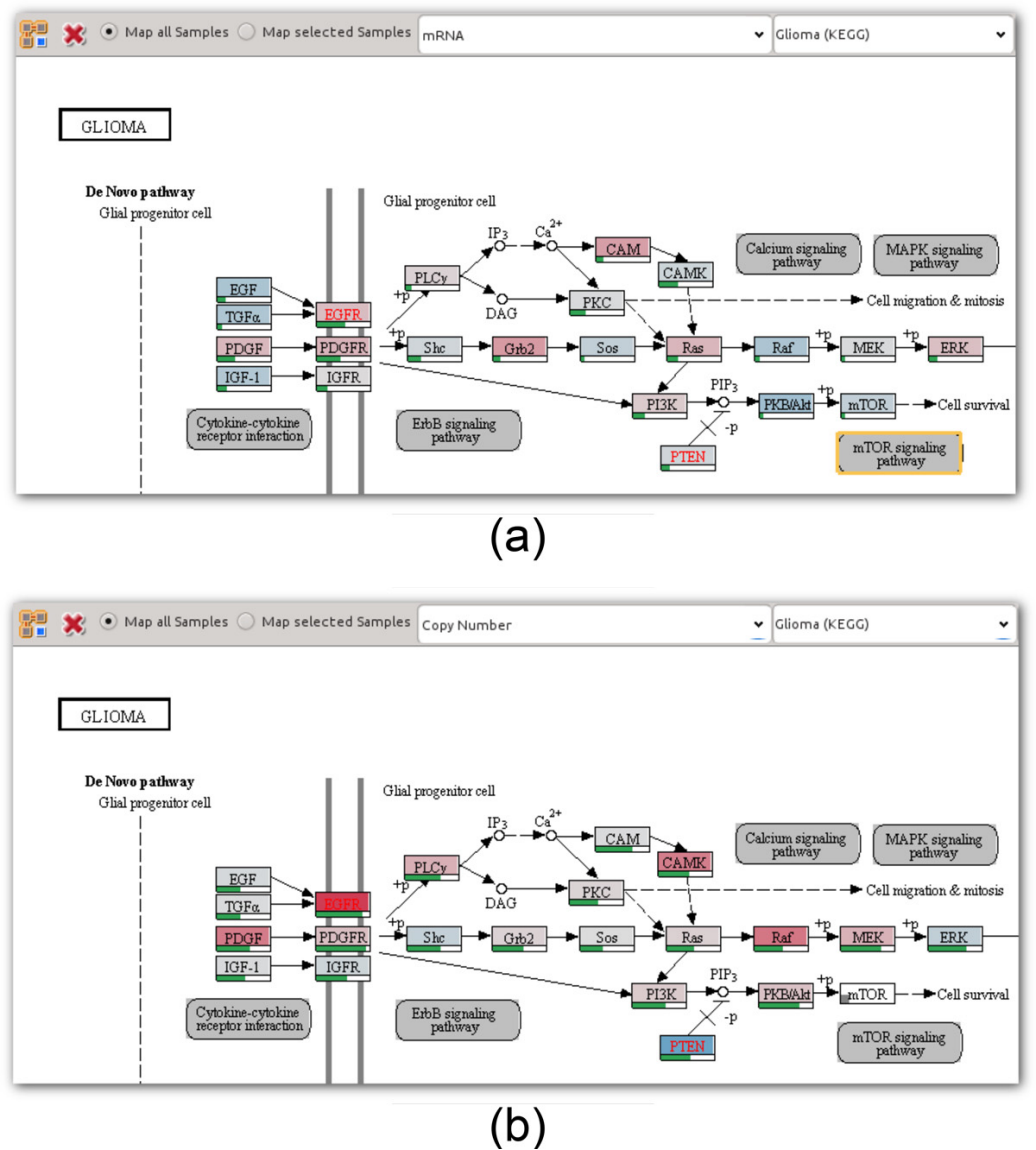
**On-node data mapping**. Averages of mapped samples for different data types of the TCGA glioblastoma dataset overlaid as color codes on nodes of the KEGG *glioma *pathway. Bars at the bottom of the nodes encode the variance across the mapped samples. (a) mRNA data, using a blue-white-red color map where blue corresponds to under-, white to regular, and red corresponds to overexpression. (b) Copy number data, also on a blue-white-red color map, where blue corresponds to deletions, white to a regular copy number, and red to increased copies of the gene.

To address the overview task where analysts want to get a rough indicator of the mapped experimental values, we calculate the average of all experimental sample values and multi-mappings, if applicable, and color-code the nodes accordingly. If multiple data types are available, the analyst can choose which of them should be mapped. Figure 5(a) shows the *Glioma *pathway with on-node mappings of mRNA data, while Figure 5(b) shows the same pathway overlaid with copy number data.

For numerical and ordinal data we use a blue-white-red color map. We decided to use white as a neutral base of the color map to be able to intuitively represent data that has a neutral base, as, for example is the case with copy number data, which has a "normal" status. In addition, the blue-white-red color map avoids the drawbacks of the common red-black-green color map for red-green color blind users. A two-color gray-red color map is used for nominal data with two categories, such as mutation status data. To indicate cases where experimental data is missing, we show a small rectangle in the lower left corner of the node, as can be seen, for example, in the *mTOR *node in the lower right part of Figure 5(b).

Since the aggregation of all samples and possible multi-mappings into an average value hides all variation, we additionally provide the standard deviation encoded as a green bar below each node, as shown in Figure 5. This indication of variance is very valuable for the overview task. High variation (corresponding to an almost full bar), as can be seen for instance for the *PDGFR *gene in Figure 5(b), is an indicator for potentially interesting experimental data that is worth to be investigated in detail using the enRoute view.

### enRoute view

Once a path has been selected in the pathway view, it can be analyzed in detail in context of experimental data in the enRoute view. The path is displayed in a linear, top-down layout, which is ideally suited to show rows of experimental data (*data rows*) right next to the nodes they are associated with. As a node can have multiple mapped data rows, we adapt the spacing between nodes of the path so that all rows can be shown with a uniform height. Such multi-mappings or the occurrence of complex nodes (nodes that consist of multiple subnodes) in the path make it very hard, if not impossible, to determine which data row belongs to which node using their position alone. Therefore, we connect each node with corresponding data rows using ribbons, as shown in Figure 2. To make the association between data rows and nodes even more obvious, we alternate the shade of gray in the data rows' backgrounds for each node. Figure 11(b) illustrates an example where these alternating shades of gray allow us to disambiguate the mappings of multiple subnodes of a complex node to corresponding data rows.

Following the divide-and-conquer visualization strategy [27], we group experimental data in the enRoute view based on a homogeneity criterion. For example, experiments can be grouped by the species they belong to (homogeneity with respect to semantics), or a grouping can be obtained by clustering (homogeneity with respect to statistics). As illustrated in Figure 2, the groups are depicted as columns resulting in an overall tabular layout. We address the *heterogeneity requirement (R II) *by allowing the individual groups to originate from different datasets. However, all experiments within a group must be from a single dataset.

#### Visualizing the path

**Figure 6:**
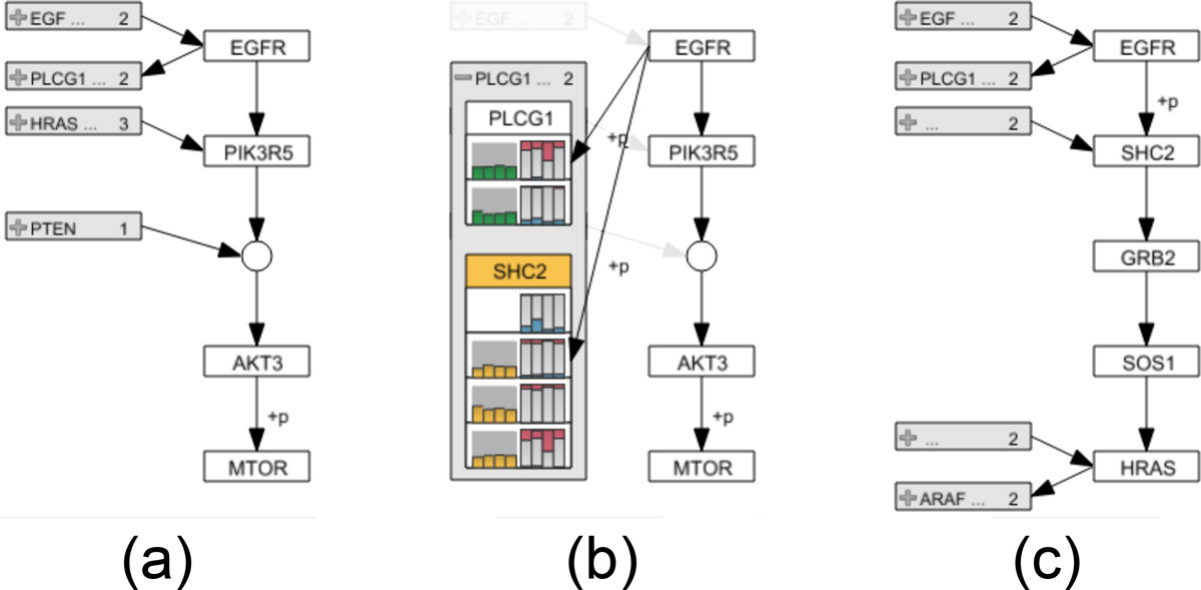
**Path representation and branch switching in the enRoute view**. (a) The extracted path from the node *EGFR *to *MTOR *is shown top-down along with branches on the left. (b) Expanding the abstract node for leaving branches of *EGFR *reveals the individual branch nodes *PLCG1 *and *SHC2*, which show previews of associated experimental data. (c) By selecting *SHC2 *the associated branch replaces all path nodes below *EGFR*. All nodes of the branch are added up to the point where the branch is no longer unambiguous. In this case *HRAS *represents the end point, as it has two leaving branches.

In addition to showing the extracted path top-down in the enRoute view, we also display branches that join or leave the path in order to preserve some of the topological information present in the pathway maps. We indicate a branch by showing its first node relative to the node where the branching occurs in the extracted path. In order to maintain a compact path representation, multiple branches that join or leave a single node of the path are abstracted into expandable nodes, one for all joining and one for all leaving branches, as shown in Figure 6(a). These abstract branch nodes indicate the number of branches they represent and also show labels for them, if sufficient space is available. Abstract branch nodes can be expanded at any time to reveal the individual branch nodes, which display previews of associated experimental data, as shown in Figure 6(b). When expanding a node, its content is rendered on top of the other branches, which are grayed out.

As illustrated in Figure 6(c), an analyst can interactively switch to a branch by selecting the corresponding branch node. A selected branch replaces all nodes in the extracted path above or below the node where the branching occurs, depending on whether it is a joining or leaving branch. All nodes of the branch are added to the path until either a new branch or a dead end is reached. As the enRoute visualization technique synchronizes all corresponding elements among its components, any changes to the path caused by branch switching are propagated back to the pathway view, thus keeping the highlights of the selected path up-to-date. Also, the synchronization of node highlights facilitates the association of branches shown in the enRoute view with corresponding branches in the pathway maps.

#### Visualizing experimental data

Being able to display large amounts of heterogeneous experimental data is an integral part of the enRoute visualization technique (see requirements *R I *and *R II*). enRoute supports the visualization of quantitative, ordinal, and binary categorical data. As previously mentioned, we organize experimental data in rows and columns. Each row shows data that maps to a certain node in the path and columns group the data by a homogeneity criterion. Different groups may also have overlapping experiments. The captions of the individual groups are displayed at the top and at the bottom of the corresponding columns. Their background color indicates the dataset they belong to. For example, in Figure 1(b) the background of groups showing mRNA expression data is turquoise, whereas the background of copy number data groups is blue and the background for mutation data is light violet.

In molecular biology, heat maps are the standard way to visualize quantitative and ordinal data. However, it is well known that hue or value are inferior to other encodings with respect to communicating changes in the data. For both quantitative and ordinal data, encodings in position are a better choice and for quantitative data, length encodings are also superior [28]. Recently, Meyer et al. [23] also showed that a mirroring effect in expression data was much more apparent when it was visualized using line plots compared to when using heat maps. Heat maps or any other pixel-based visualization techniques are superior in terms of space efficiency and therefore scalability. enRoute, however, only requires the visualization to be scalable with respect to experiments, since the number of genes is typically small, as it is limited by the number of nodes in the path. Therefore, we prefer bar charts over heat maps for the representation of quantitative data as well as for ordinal data.

**Figure 7:**
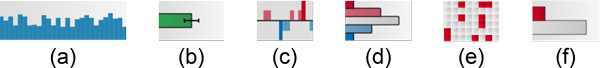
**Six visual encodings for different types of experimental data**. (a) One vertical bar is shown for numerical data point. (b) A group of numerical data points is abstracted into one horizontal bar with error bars. (c) Redundant encoding using color and length for copy number data. Red bars pointing upwards indicate an increased number of copies, whereas reduced copy numbers are shown as blue bars pointing downwards. (d) Several copy number values are abstracted into a histogram. (e) Matrix visualization for mutation status data. Red cells indicate samples where the gene is mutated. (f) Histogram abstracting the binary mutation status of the gene across samples.

In the bar charts used for quantitative data, each bar represents one value of a single experiment, as shown in Figure 7(a). In order to make the borders of adjacent bars apparent without having to waste space for drawing outlines, we color the bars using a gradient from left to right. As shown in Figure 1(b), tooltips are used to show the numerical values of the underlying data. In some cases it might be desirable to see an abstract and more compact visualization of a group of quantitative data. For this purpose, we use one horizontally aligned bar that represents the mean value of a group together with error bars, encoding the standard deviation, as shown in Figure 7(b). In contrast to the detailed representations, where the width adapts to the number of experiments in the group and available display space, the width of abstract group representations is fixed. This constant width and the horizontal alignment of the abstract bars allows analysts to compare values of the same group across rows along the path more easily. However, for tasks that require comparisons across multiple groups, the detailed representation with vertical bars are preferable.

As copy number data commonly occurs either in ordinal or quantitative form, we use an optimized encoding that can deal with both of them. Ordinal copy number data is often categorized into *high *and *low *increase of gene copies, a *normal *copy number, *deletion on one allele*, and *deletion on both alleles*. As shown in Figure 7(c), our encoding of this data redundantly uses the length, color, and orientation of bars. For highly increased copy numbers, we show long, dark red bars pointing upwards from a base line. For low increases we use shorter, light red bars. Similarly, deletions are represented by dark and light blue bars pointing downwards. No bar is shown for normal copy numbers. The same encoding can be used for quantitative copy number data. The higher the increase in copies, the longer and darker the red bar is. The same concept applies to deletions. Just like for general quantitative data, we also employ an abstract representation for groups of copy number values. As shown in Figure 7(d), we use a horizontal histogram, which makes use of the same color coding as the detailed copy number representation.

For binary categorical data, such as data on whether a gene is mutated or not, we use a matrix visualization where each cell corresponds to a sample, as shown in Figure 7(e). For the mutation status example we color samples that are mutated in red, while non-mutated samples are shown in the background color. While the matrix layout deviates from the convention used for numerical and ordinal data of placing all samples side-by-side, we found it to be significantly more space-efficient compared to presenting mutation data in line with the bar-techniques. Space efficiency is important for mutation data since mutated genes are scarce in many datasets. Also, since only binary information is encoded, the redundant encoding using length and color is obsolete. For the abstract summary representation we use a histogram, similar to the one used for copy number data as shown in Figure 7(f).

The previously mentioned data previews, shown on-demand for branch nodes, use an encoding similar to the abstract data representations, as can be seen in Figure 6(b). For each group of mRNA data one bar indicating the group's mean value is drawn. For copy number and mutation data, we show one stacked bar per group.

The enRoute visualization technique makes use of synchronized highlighting of corresponding elements across all its components but also within all components. The latter case is especially useful in the experimental data display. By highlighting a set of experiments in one group, we allow analysts to identify these experiments in other groups, even for different data types. For example in Figure 10(b), all cell lines with an increased copy number are highlighted, which allows analysts to relate the increase in copy number with mRNA expression. As evident in this figure, scattered selections make it difficult to quantify the number of selected experiments. To alleviate this problem, we add tooltips to the groups' captions showing the total number of experiments and the number of currently selected experiments of each group.

### Choosing experimental data and groupings for enRoute

**Figure 8:**
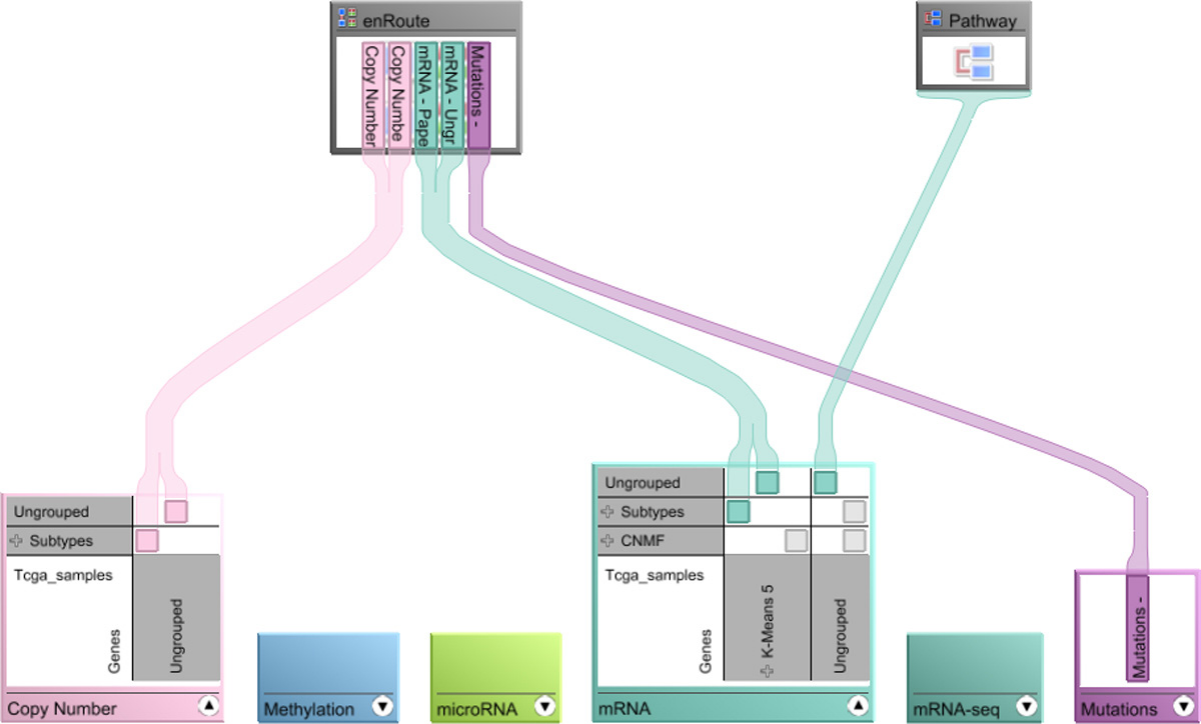
**Assignment of experimental data and groupings to the enRoute view using Caleydo's Data-View Integrator**.

Up to this point, we have assumed that decisions on which datasets and which groupings of the datasets to show are already made. However, given a large set of datasets and a variety of alternative groupings to choose from for every dataset, this presumably easy task is in fact not trivial. To support analysts in the task of selecting datasets and groupings and assigning them to views, Caleydo provides a dedicated view, the *Data-View Integrator (DVI) *[15]. As shown in Figure 8, the DVI view uses a graph representation that shows all loaded datasets at the bottom and all open views at the top. Each dataset is associated with a unique color that is the same as the one used in the background of the dataset labels in the enRoute view. For tabular datasets it is quite common to have groupings, such as clusterings, of both rows and columns available. These alternative groupings are represented in the matrix layout shown when exploring a dataset in detail, where alternative row groupings are shown in the rows and column groupings in the columns. An analyst can assign grouped datasets to enRoute by dragging the blocks onto the view representation at the top. Connection bands between datasets and views help analysts to understand the association of the data to the view. This is in particular helpful for highly heterogeneous configurations with multiple datasets, groups, and views. Users can switch to the DVI view at any time during a pathway analysis in order to refine the mapped experimental data. Additionally, new datasets and groupings can be added at runtime.

## Implementation

The approach presented in this paper is implemented as a module of **Caleydo**, which is an open source visualization framework for biomolecular data [17]. Binary versions for Windows, Linux, and Mac, as well as the full source code and help material are available at http://www.caleydo.org. Both, our visualization technique and the visualization framework are implemented in Java and OpenGL using the freely available JOGL library (available at https://jogamp.org/jogl). In order to highlight selected pathways, we make use of a free implementation of the Bubble Sets technique [29] that we integrated into the Caleydo framework.

Caleydo allows analysts to import data sets with large amounts of experiments, such as mRNA, copy number, and mutation status datasets. We provide pre-packaged and preprocessed TCGA datasets (see http://tcga.caleydo.org), which contain up to 900 samples (depending on the subtype stratification) and *~*20,000 genes each. As demonstrated in Figure 10, our visualization technique scales well to such large datasets. To cope with the limitations of available screen space, we make use of scroll bars for longer path in enRoute, which is justified by the linear nature of the exploration process along the path.

Our current implementation builds upon the well-established KEGG and Wikipathways databases, which provide information about pathways as image data in combination with an XML-based descriptions of the graph. The description includes the topological information as well as the position and the size of nodes. We use this information to render the various augmentations described on top of the pathways.

**Figure 9:**
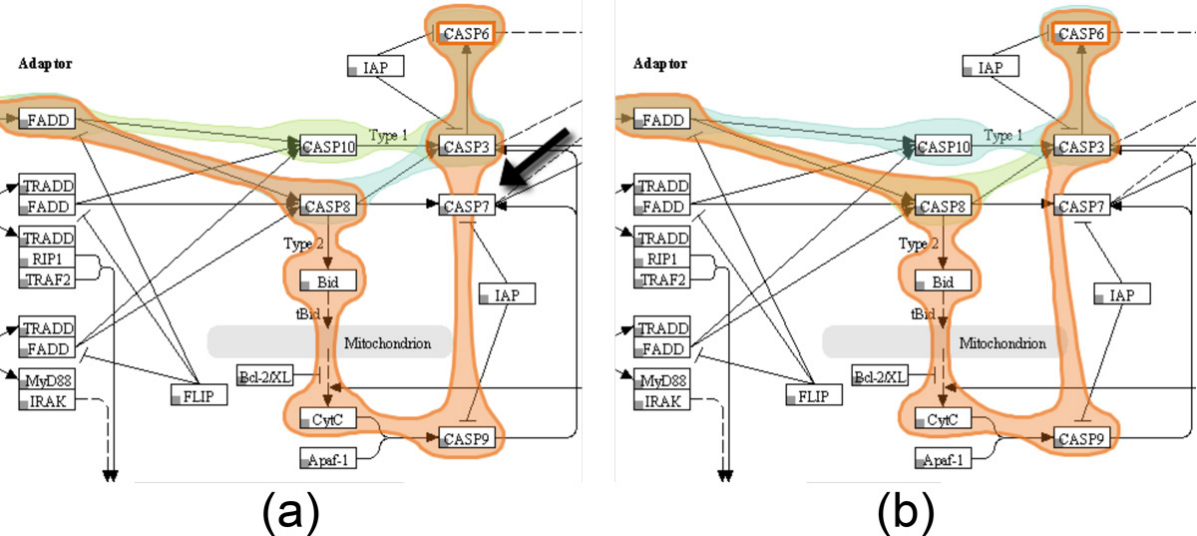
**Re-routing of Bubble Sets for path highlights**. (a) As the pathway description does not provide information about the routes used to connect the nodes, a collision of the path leading form *CASP9 *to *CASP3 *with the unrelated node *CASP7 *occurs. (b) To avoid this, we redirect the path around the node.

Since pathway databases such as KEGG do not provide information about how edges are routed in the images, directly connecting nodes in a path using straight edges can lead to overlaps between edges and nodes that are not part of the path. In Figure 9(a), for example, the edge highlight between node *CASP9 *and *CASP3 *collides with the representation of node *CASP7*, which is not a member of the selected path. In such cases, we route edges around nodes to avoid overlaps. Figure 9(b) shows the Bubble Set with a refined route from *CASP9 *to *CASP3*.

The Bubble Sets algorithm renders the overlays semi-transparent on the top of an existing base representation. However, if the base representation uses color-coding, overlaying colored Bubble Sets can lead to a wrong interpretation of the encoded information, as the colors of the Bubble Sets interfere with the color of the augmented content. Additionally, blending decreases the legibility of the original content. To avoid this problem, we cut the area of nodes out of the Bubble Sets.

Pathways can be selected from a drop-down list, which contains all loaded pathways. The full list is reduced on-the-fly to only contain the ones that match a user-specified query string. In addition to the title-based search, analysts can click embedded pathways to replace the current pathway with the full version of the embedded one.

## Case studies

We developed the enRoute visualization technique in close collaboration with a biologist from the Medical University of Graz, who is also an author of this paper. To evaluate the utility of the enRoute visualization technique, we conducted two case studies using different datasets together with this biologist. The first dataset is taken from the Broad-Novartis Cancer Cell Line Encyclopedia (CCLE, http://www.broadinstitute.org/ccle/home), which contains the genetic and pharmacologic characterization of a large panel of human cancer cell lines. The second is a microarray dataset from a model of hepatocellular carcinoma collected at the Medical University of Graz, which can be found through the corresponding original article [30].

### Apoptosis regulation in cancer cell lines

The first case study investigates the difference in regulation of the apoptosis pathway in different human tumor cell lines from various organs. Apoptosis is the programmed death of a cell due to internal damage or as a consequence of external stimuli and involves a signaling cascade, which is not mediated by phosphorylation but by targeted degradation of proteins through enzymes called caspases. To initiate apoptosis in a cell, a ligand called *TRAIL *or *TNFa *binds to receptors on the outer cell surface. This leads to activation of receptor associated death domain containing proteins, which activate the first of a cascade of caspases, which in turn eventually leads to the cleavage of proteins in the cell subsequently triggering apoptosis. During apoptosis the cell shrinks in volume, exhibits nuclear fragmentation, chromosomal DNA fragmentation, and release of Cytochrome c from the mitochondria, which eventually leads to cell death.

**Figure 10:**
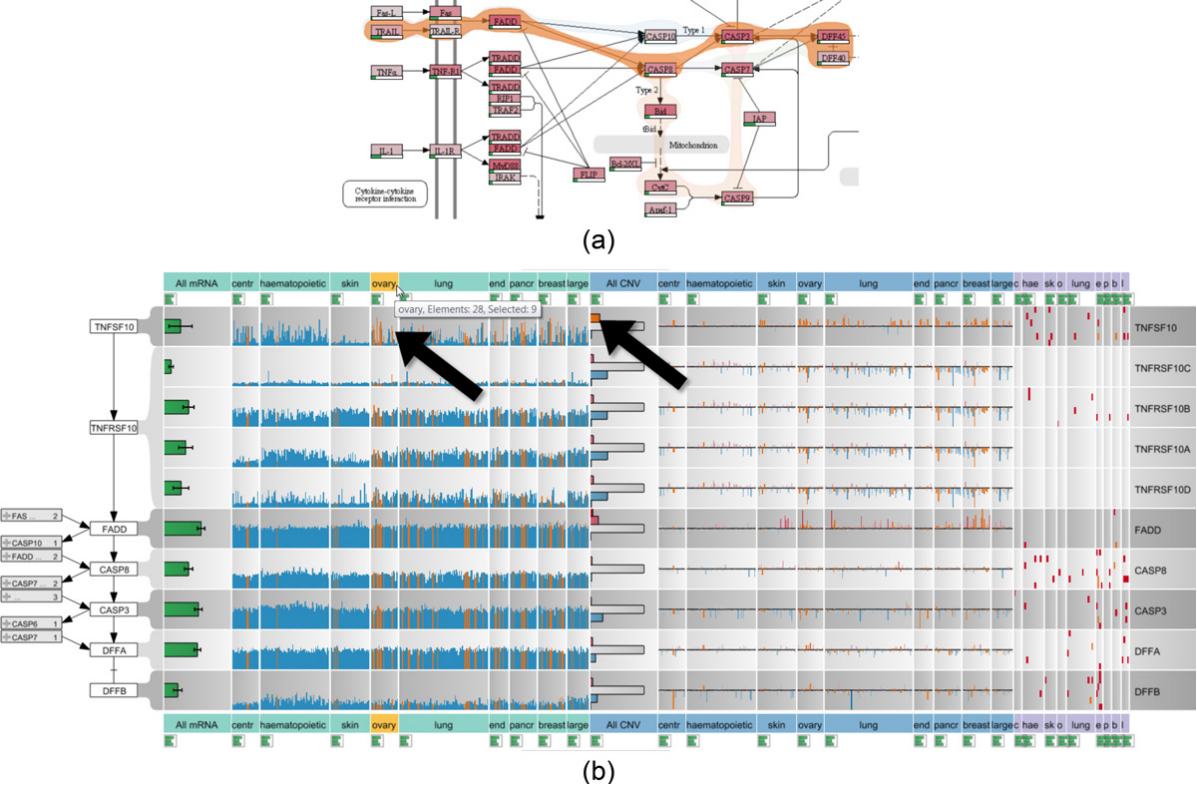
**Analysis of CCLE experimental data in context of apoptosis**. (a) Different paths between the nodes *TRAIL *and *DEF40 *are highlighted in the *Apoptosis *pathway map. The orange Bubble Set shows the chosen path. (b) The selected path is shown in context of associated mRNA expression, copy number, and mutation status data from the CCLE dataset with about 350 samples each. As indicated by the arrows, highlighting all samples with an increased copy number of *TNFSF10 *reveals the correlation with increased expression levels in the ovary cell lines. Notice that while the labels shown in the enRoute view are not identical to the labels in the pathway view, the labels are homologous and deviate since pathway databases use one of many aliases for genes or proteins.

As first step of the analysis, the *Apoptosis *pathway of the KEGG database is loaded into the the pathway view. The researcher selects *TRAIL *as the starting point and *DEF40 *as the endpoint of the signaling cascade to be investigated. As shown in Figure 10(a), the system automatically highlights all possible paths from *TRAIL *to *DEF40 *and allows to switch between these alternative paths. The selected path is extracted and displayed in a linear layout in the enRoute view, which shows detailed CCLE mRNA expression, copy number variation, and mutation status data for the genes that map to the nodes of the path, as can be seen in Figure 10(b). In this example, the grouping of the experimental data reflects the source organ of origin of the cell lines. The analyst can now easily identify differences in the copy number variation, gene expression, and mutation status for these groups and relate them immediately to the stages of the linearized path, which would not be possible using other techniques. As indicated by the upward pointing bars for the copy number data, there is a clear amplification of *TNFSF10 *(an alias for *TRAIL*) for cell lines originating from ovary, lung, or breast tissue among others. By selecting all samples with an increased copy number of *TNFSF10 *in the histogram, the system also highlights these cell lines in the mRNA expression plots, as indicated by the arrows in Figure 10(b). The analyst can now confirm that ovary cell lines with a higher copy number of this gene also show a higher expression of *TNFSF10 *in the mRNA expression plots. Based on this data, one could interpret that these cell lines should be prone to apoptosis, as they have high amounts of the cytokine triggering the cell death. However, given the nature of these cell lines which are derived from human tumors, which are by definition resistant to apoptosis, this conclusion would be misleading. An explanation for this seemingly strange behavior can be found by looking at the copy numbers of the genes *TNFRSF10A *to *TNFRSF10D*, which are receptors for *TNFSF10 *cytokine, further downstream the path. Here, it quickly becomes evident that these genes exhibit a frequent loss of copies, which is easy to see when looking at the many blue bars pointing downwards for these genes. This explains how these cells can still form a tumor, as the loss of the receptor makes the cells immune to the stimulus by the *TNFSF10 *cytokine, thus preventing apoptosis initiation.

In summary, enRoute enabled the analyst to study copy number variation, gene expression, and mutation status of a large number of samples in parallel in a clearly visualized and linearized sub-path of the complex network initiating apoptosis. The researcher stated that the linearization of the signaling cascade and the clear association of the other data entities to the individual steps of this cascade are very intuitive and greatly facilitated the interpretation of the data and the deduction of a biological interpretation from the dataset.

### HCC xenograft models

**Figure 11:**
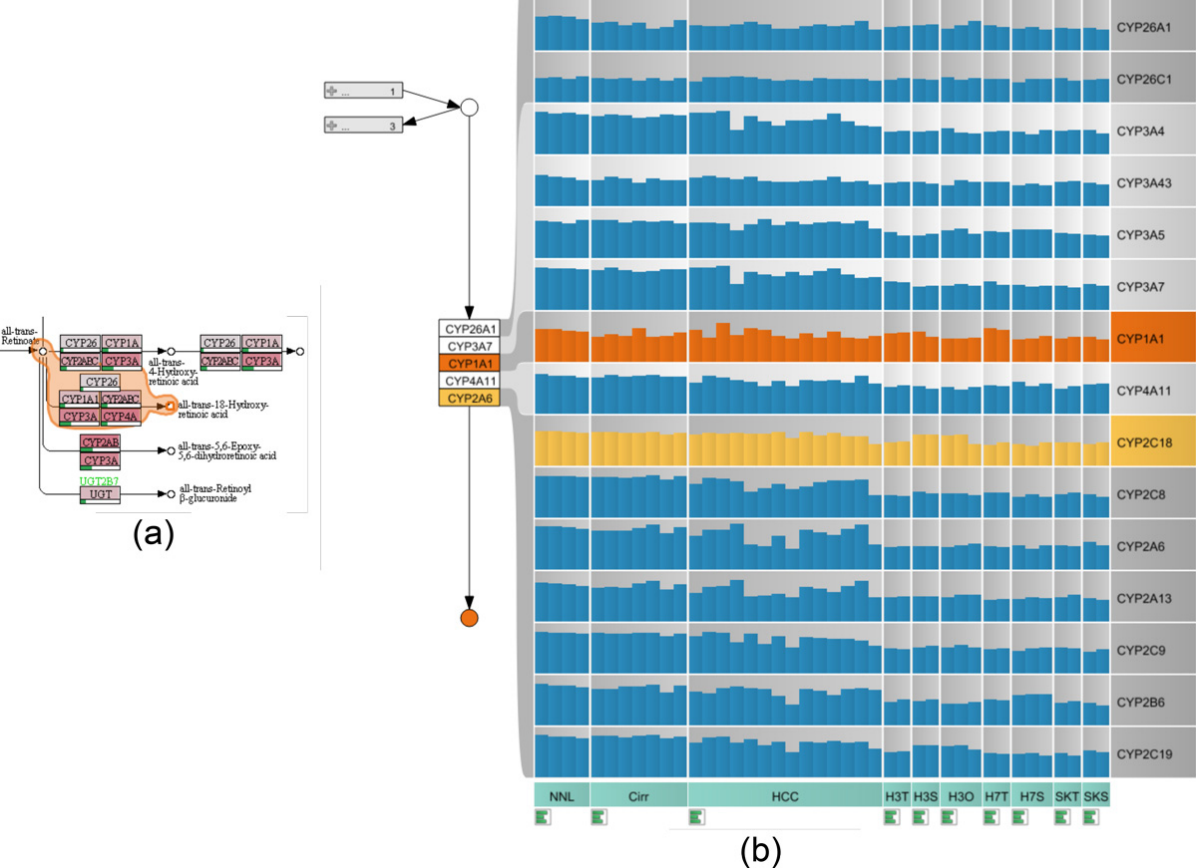
**Resolving multi-mappings in the KEGG pathway map *Retinol Metabolism***. (a) A path that contains a complex node with many multi-mappings is selected. (b) This path is shown in the enRoute view, together with gene expression data for normal liver (NNL), cirrhosis (Cirr), hepatocellular carcinoma (HCC), and three cell line models, Hep3B, HUH7, and SK-Hep in the conditions Hep3B tissue culture (H3T), Hep3B subcutaneous graft (H3S), Hep3B orthotopic graft (H3O), HUH7 tissue culture (H7T), HUH7 subcutaneous graft (H7S), SK-Hep tissue culture (SKT), and SK-Hep subcutaneous graft (SKS). *CYP2C18 *(yellow) is highly expressed in the Hep3B model and *CYP1A1 *(orange) is upregulated in HUH7.

The second use case demonstrates the ability of the enRoute path extraction feature to aid in a common problem when interpreting pathway maps. In biological reaction systems enzymatic reactions are often carried out not by a single gene or protein but by a family of proteins encoded by several genes. Additionally, enzymatic reactions are often not specific to a single protein family, but can be substituted by other enzymes, which, however, often work with different efficiency. This biological diversity leads to functional nodes in pathways that contain a multitude of genes with historically designated gene names often not indicative of the real function of these genes. It is extremely difficult for the researcher to keep in mind which genes are behind a single node of a pathway and thus understand the mapped biological data. Cross referencing of expression levels to genes and functional nodes can thus only be achieved by resolving the multi-mapping in a node to gene names which can then be mapped to the individual gene expression levels. In enRoute, the researcher can select nodes upstream and downstream of the enzymatic reaction, which is then resolved in a linearized representation of all genes involved. An example is the conversion of *all-trans-Retinoate *to *all-trans-18-Hydroxy retinoic acid*, which is a reaction contained in the *Retinol (Vitamin A) metabolism *pathway shown in Figure 11(a). The single node that represents this reaction contains 16 different proteins or genes that are involved. As depicted in Figure 11(b), enRoute resolves this node into a convenient map of genes, thus making it possible to map the gene expression of each individual gene in all experimental conditions of this experiment. The dataset mapped onto the Retinol pathway was generated by gene expression profiling of normal and cirrhotic human liver, hepatocellular carcinoma, and grafts of three human tumor cell lines (Hep3B, HUH7, and SK-Hep) into immunodeficient mice. Expression was measured in liver samples from patients and in samples from tumor cells grown in culture (TC), as subcutaneous grafts (SC), or as orthotopic grafts (Ortho) in the liver of experimental animals. The task was to find out how well the cell line models correlate to the human disease, with special focus on drug metabolism and oxidative stress. The response of tumors to anticancer treatments is closely linked to the activity of *cytochrome p450 *enzymes, which metabolize drugs and mediate oxidative stress. When studying the aforementioned conversion of retinoate to retinoic acid by cytochrome p450 enzymes, it can now be seen that the node *CYP2A6 *actually contains members of the whole *CYP2 *family of genes and that the expression of these genes is uniform in normal liver and cirrhosis, but very variable in HCC, dividing these cases into a low expressing and a high expressing group. It is immediately visible that the xenograft models are only representative of the low *CYP2 *expressing group of HCCs. Decomposition of the complex nodes allows the identification and investigation of the individual expression patterns of genes contained in the complex node. It becomes obvious that the expression of *CYP2C18 *(highlighted in yellow), contained in the node labeled *CYP2A6*, is higher in the Hep3B model than in the other cell lines.

Additionally, it can be detected that *CYP1A1 *is highly expressed in HUH7 tissue culture cells. All this information was not visible to the researcher using conventional on-node mapping approaches and was successfully visualized using enRoute. Node decomposition is an integral feature of the enRoute path extraction and thus makes the association of many mapped nodes and their corresponding experimental data readily available to the researcher.

## Conclusion and future work

Enabling the joint analysis of biological pathways and large amount of experimental data by means of visualization is a challenging problem. In this paper, we provide a list of requirements that have to be met to support such analysis. We introduce the enRoute visualization technique, which addresses these requirements using a tightly-coupled dual-view approach. Our visualization technique allows experts to select a single path from a pathway map, which is then extracted and shown in linear form in a second view, the enRoute view. This view allows experts to investigate associated experimental data in detail by displaying it in a tabular layout right next to the extracted path. The conducted case studies using two different datasets confirmed the utility of our visualization technique.

Although we currently already support pathways from both the Wikipathways and the KEGG database, we plan to integrate further resources for biological networks. For instance, an integration of the EBI IntAct database [31] will make the analysis of protein interaction networks possible using enRoute.

enRoute allows to select nodes independent of the structure of the pathway graph. However, the boundaries of a single pathway graph also limit the potential of enRoute. Consequently, we plan to extend enRoute to also support the analysis of paths spanning multiple pathways, which introduces new challenges for scalability concerning the topology of the graph. Solving these problems is subject of future research. Another interesting area of research is the selection of pathways, which we have only briefly touched in this paper. We intend to implement common measures for identifying interesting pathways in the context of the available experimental data and integrate them in a unified data and pathway visual analysis workflow.

## Competing interests

The authors declare that they have no competing interests.

## Authors' contributions

CP, AL, MS, DK, and DS developed the concept of the enRoute visualization technique. CP, AL, MS, and DK implemented the Pathway view and the enRoute view for the Caleydo visualization framework. KK consulted on biological relevance and evaluated the visualization technique with regard to its applicability for biological data analysis. All authors contributed to this manuscript.

## Declarations

This publication was funded by the IEEE Symposium on Biological Data Visualization (BioVis) as a supplement of highlights.

The articles in this supplement have undergone the journal's standard peer review process for supplements. The Supplement Editors declare that they have no competing interests.

This article has been published as part of *BMC Bioinformatics *Volume 14 Supplement 19, 2013: Highlights from the 2nd IEEE Symposium on Biological Data Visualization. The full contents of the supplement are available online at http://www.biomedcentral.com/bmcbioinformatics/supplements/14/S19.
